# Using the National Mental Health Service Planning Framework to inform integrated regional planning: a case study in Tasmania, Australia

**DOI:** 10.1186/s13033-023-00591-w

**Published:** 2023-07-22

**Authors:** Kate Gossip, Claudia Pagliaro, Charlotte Comben, Kevin Fjeldsoe, Harvey Whiteford, Sandra Diminic

**Affiliations:** 1 Queensland Centre for Mental Health Research The Park - Centre for Mental Health, Locked Bag 500, Sumner Park, BC, QLD 4074 Australia; 2 School of Public Health, The University of Queensland, Brisbane, QLD Australia

**Keywords:** Mental health, Mental health services, Health service planning, Service mapping, NMHSPF

## Abstract

**Background:**

The aim of this study was to demonstrate the application of a needs-based mental health service planning model in Tasmania, Australia to identify indicative directions for future service development that ensure the equitable provision of mental health services across the State.

**Methods:**

The activity and capacity of Tasmania’s 2018–19 mental health services were compared to estimates of required care by: (1) generating estimates of required care using the National Mental Health Service Planning Framework (NMHSPF); (2) collating administrative mental health services data; (3) aligning administrative data to the NMHSPF; and (4) comparing aligned administrative data and NMHSPF estimates to identify priority areas for service development. Findings were contextualised using information about service location, population demographics, and upcoming service development.

**Results:**

Bed-based services capacity reached 85% of the NMHSPF estimate. However, access to certain bed types was inequitable across regional areas. Access to jurisdictional clinical ambulatory team-based services was lowest in the South, while overall full-time equivalent staff capacity reached 58% of the NMHSPF estimate. Access to Primary Health Tasmania (PHT) primary care services was highest in the North West; access to Medicare services was highest in the South. Collectively, activity across primary care (PHT, headspace and Medicare) reached 43% of the NMHSPF estimate. Over half of Community Managed Mental Health Support Services were state-wide services.

**Conclusions:**

This study demonstrates the application of a needs-based planning model for mental health services. Findings revealed service priority areas across Tasmania and highlight considerations for needs-based planning.

**Supplementary Information:**

The online version contains supplementary material available at 10.1186/s13033-023-00591-w.

## Background

Mental illness cannot always be treated by one type of service [1]. However, there has traditionally been a lack of coordination between services and sectors of the mental health system. Internationally, a history of marginalisation of mental health and mental health care, as well as ad-hoc policy implementation, has resulted in the fragmented governance of mental health systems, leading to gaps in service delivery and care [2, 3].

In Australia, the country’s Fifth National Mental Health and Suicide Prevention Plan (Fifth Plan) acknowledges the need for more coordinated approaches to mental health service planning [4]. The Fifth Plan therefore prioritises the joint development of integrated, regional mental health and suicide prevention plans by Primary Health Networks (PHNs) and Local Hospital Networks (LHNs). PHNs are funded by the federal government to plan and commission clinical ambulatory and psychosocial support services (i.e., non-clinical services to support recovery/community participation), and LHNs are funded by state and territory governments to: (1) plan and provide jurisdiction-funded specialised clinical bed-based and ambulatory services, and (2) fund psychosocial support services often delivered by non-government organisations [5, 6]. PHNs and LHNs typically operate within the same overlapping geographical boundaries. Separately, the federal government also funds population-specific services (e.g., via the Department of Veterans Affairs and National Disability Insurance Scheme (NDIS)), and other clinical services via the Medicare Benefits Schedule (MBS)). Care is also available from private hospitals, and private health insurance can be used for bed-based and clinical ambulatory care. With many funders and providers, it is often difficult to quantify the current mix and level of services available to inform the development of joint regional mental health plans within a planning region.

Understanding mental health service provision is integral to evidence-based planning [7]. Several data collections and/or frameworks can be used to describe and quantify available services; these include: nationally agreed minimum datasets [8]; the World Health Organization’s Mental Health Atlas [9]; and the Description and Evaluation of Services and Directories in Europe for Long-Term Care (DESDE-LTC) classification system [10]. Each of these data collections/frameworks allow for like-for-like comparisons of service provision across regional areas, jurisdictions and/or countries. However, a more informative analysis involves comparing current service provision against service provision required to meet population needs [7].

The National Mental Health Service Planning Framework (NMHSPF) is a national average needs-based planning model that produces estimates of the resourcing required to deliver mental health care in Australia [11]. It provides a common language for the Australian mental health system and an integrated structure that enables planners from different sectors (e.g., from primary care to specialist services) to map current service provision against estimates of required care [12]. Differences between on-the-ground service provision and these estimates can provide indicative directions for service development. The NMHSPF encompasses individually tailored services funded by the Australian mental health system and therefore does not produce estimates for: self-help and population-based programs, including crisis help-lines; school-based supports funded by the education system; employee or workplace-based supports; general community and social services (e.g. child protection); drug and alcohol and general physical health care services. The complementary national Drug and Alcohol Service Planning (DASP) model provides resource estimates for substance use services in Australia [13]. While people with comorbid disorders will be counted in both models, end users need to consider the best way to provide integrated care for these populations, which may include dual diagnosis services. Further information on the NMHSPF model is available on the NMHSPF website [14].

The NMHSPF has been used to inform regional service planning in a number of Australian catchments [15–20], to support the development of jurisdictional mental health plans [21–23] and for broader reform work [24, 25]. The NMHSPF was built on earlier needs-based mental health planning models in Australia [26], with an expanded scope and user base. Similar needs-based service planning models have been developed internationally, such as a model to estimate demand for substance use treatment in Canada [27] and for the mental health workforce in low- and middle-income countries [28].

In 2019, the Tasmanian PHN (Primary Health Tasmania (PHT)) and the Tasmanian State government Department of Health (DoH) commissioned a project to apply the NMHSPF to inform regional mental health service planning. Tasmania is Australia’s only island state, located 240 km to the south of the Australian mainland. It has the oldest population in Australia which is ageing faster than any other jurisdiction due to internal migration [29]. Tasmania is also Australia’s smallest and poorest state [30, 31]. Collectively, these factors have presented unique challenges when it comes to the planning of mental health services that meet the needs of all Tasmanians.

The state’s existing mental health plan, Rethink Mental Health, was published in 2015, prior to the establishment of PHNs and the release of the Fifth Plan [4, 32]. At the mid-point of Rethink’s 10-year vision, the Tasmanian DoH and PHT required an understanding of how mental health service provision was tracking. The purpose of this research project was to therefore identify indicative directions for future service development that ensure the equitable provision of mental health services, at the regional level, across the State of Tasmania. This was to be done by analysing current service accessibility, activity, and resourcing and comparing the latter two to NMHSPF estimates. Specifically, the project aims were to:

(1) Map the current distribution of mental health services access, activity and capacity in Tasmania;

(2) Undertake a comparative analysis between on-the-ground service provision in Tasmania and the NMHSPF estimates of optimal activity and capacity; and

(3) Identify considerations for others who may undertake comparative analyses using the NMHSPF or other needs-based planning models.

## Methods

### Study context

In 2017, there were approximately 522,000 people living in Tasmania, almost half of whom resided in the capital city of Hobart in Tasmania’s South [33]. Tasmania comprises 29 Local Government Areas (LGAs), one LHN and one PHN. It is divided into three health service regions (HSRs) (South, North and North-West). The major centres of mental health service delivery across the state are scattered along the coastline of the three HSRs in the following cities/towns: Hobart (South HSR); Launceston (North HSR); and Burnie and Devonport (North-West HSR).

### Study design

This study methods followed four steps: (1) generate activity and capacity estimates from the NMHSPF-Planning Support Tool, (2) collect access, activity and capacity data from publicly available sources or data custodians, (3) transform data to align with the NMHSPF, and (4) compare transformed data to NMHSPF estimates. Analyses were conducted at the LGA level, based on consumer usual area of residence, where possible.

### Generation of NMHSPF optimal activity and capacity estimates

The NMHSPF-Planning Support Tool (V2.2) was set up to produce activity (e.g. occasions of service) and capacity (e.g. number of employed clinical FTE staff, indicative costs) estimates for each of the 29 LGAs in Tasmania for the year 2018-19. Local salary data provided by PHT and the DoH were inputted into the NMHSPF-Planning Support Tool to ensure indicative cost estimates better reflected true costs in Tasmania. All estimates were stratified by a range of variables such as consumer usual area of residence (LGA) and consumer age groups as designated in the NMHSPF.

### Collection of on-the-ground activity and capacity data in Tasmania

In-scope services included: (1) those providing individually tailored care directly to individuals with mental illness or their carers; or (2) those that undertake duties intended to improve the well-being of individuals with a mental illness (e.g. coordination of care needs). Service activity and capacity data pertaining to in-scope services were collected from administrative datasets and reports that were publicly available or requested from data custodians. Service sectors included: bed-based mental health services (i.e., bed-based services delivered in hospital and residential settings); jurisdictional clinical ambulatory services (i.e., multidisciplinary team-based services provided in outpatient and community settings); primary care and private clinical ambulatory services (i.e., clinical ambulatory services delivered in community settings including MBS services and services funded through PHT); and psychosocial support services, which are referred to as community managed mental health services (CMMHS) in Tasmania. Table 1 includes detail on the types of data available for analysis. Information on the location of services (i.e., HSR and/or LGA location) was also collected. Activity and capacity data were not included if services were established in the final three months of the 2018-19 financial year as the analysis focussed on service delivery across the entire year.

**Table 1:** Mental health services data included in the analysis, stratified by service sector^1^

Service sector	Service type	Data year^2^	Available data
Bed-based services	Tasmanian DoH funded bed-based mental health services (i.e. hospital admitted and residential services)	2018–19	• No. of consumers • Consumer usual area of residence • Acute bed separations • Available beds • Bed type^3^
	Privately funded bed-based services	2018–19	• Service location
Jurisdictional clinical ambulatory services	Tasmanian DoH funded clinical ambulatory mental health services	2018–19	• No. of consumers • Consumer usual area of residence • FTE staff counts • Treatment team type^4^
Primary care and private clinical ambulatory services	Clinical ambulatory services delivered by headspace services and not funded by the Medicare Benefits Schedule	2018–19	• No. of consumers • Service location • Occasions of service • Funder • Funding
	Clinical ambulatory services commissioned by Primary Health Tasmania	2018–19	• No. of consumers • Consumer usual area of residence • Occasions of service • Funding
	Mental health specific ambulatory services funded by the Medicare Benefits Schedule	2015–16	• No. of consumers • Consumer usual area of residence • Occasions of service • Expenditure
Psychosocial support services	Psychosocial support services commissioned by Primary Health Tasmania	2019–20	• Service description • Service location • Funding
	Tasmanian DoH funded psychosocial support services	2017–18	• Service description • Service location • Funding
	Commonwealth funded psychosocial support services	2016–20	• Service description • Service location • Funding (converted to estimated annual funding)
	Services defined as ‘core’ or ‘capacity’ supports under the National Disability Insurance Agency and provided to eligible consumers with a psychosocial disability	June 2019 quarter	• No. of consumers • Consumer usual area of residence • Support class description • Funding (i.e. average annual committed budget plan) • National average plan utilisation estimate for consumers with psychosocial disability

^1^ Refer to Supplementary Table 1 for further detail regarding available and unavailable data sources and data transformation

^2^ Refers to financial year

^3^ Refers to the type of care received (i.e. acute versus non-acute) as well as target population (i.e. child and adolescent, adult, older adult)

^4^ Refers to the team target population (i.e. child and adolescent, adult, older adult)

### Transformation of on-the-ground data and alignment with the NMHSPF optimal estimates

Firstly, available metadata and information provided by data custodians was used to group and align service contact types, bed types, and clinical ambulatory teams with those defined in the NMHSPF taxonomy [12, 34]. Second, service activity and capacity data were stratified by consumer usual area of residence (i.e., LGA) and consumer age groups to ensure alignment with the NMHSPF estimates. Where data were available or provided at area levels lower than LGA (i.e., Statistical Area Level 3 (SA3)), concordance files were used to aggregate data to the LGA level [35]. LGA level data were also aggregated together to allow for comparative analysis to be undertaken at the HSR and state level.

### Analysing service access

Service access was determined by analysing the number of people per 10,000 population who accessed each service. Rates were then compared within and across service sectors, HSRs, and LGAs.

### Comparative analyses

Comparative analyses were undertaken to examine differences between on-the-ground services data and NMHSPF estimates. A select group of indicators were chosen for the analysis based on the types of on-the-ground data available. Service activity indicators could be used to understand the volume and type of services delivered and service capacity indicators could be used to understand the amount of resourcing available to deliver services. For bed-based services, separations data were used as indicators of activity and the number of available beds indicated capacity. For ambulatory services, occasions of service were used to indicate service activity, and workforce FTE staff counts and/or funding or expenditure were used to measure service capacity (see Supplementary Table 1 for information on all data of interest and analysis conducted). Activity and capacity data were compared to NMHSPF estimates, stratified by LGA, HSR, and service sector.

## Results

### Location and distribution of services

**Fig. 1:**
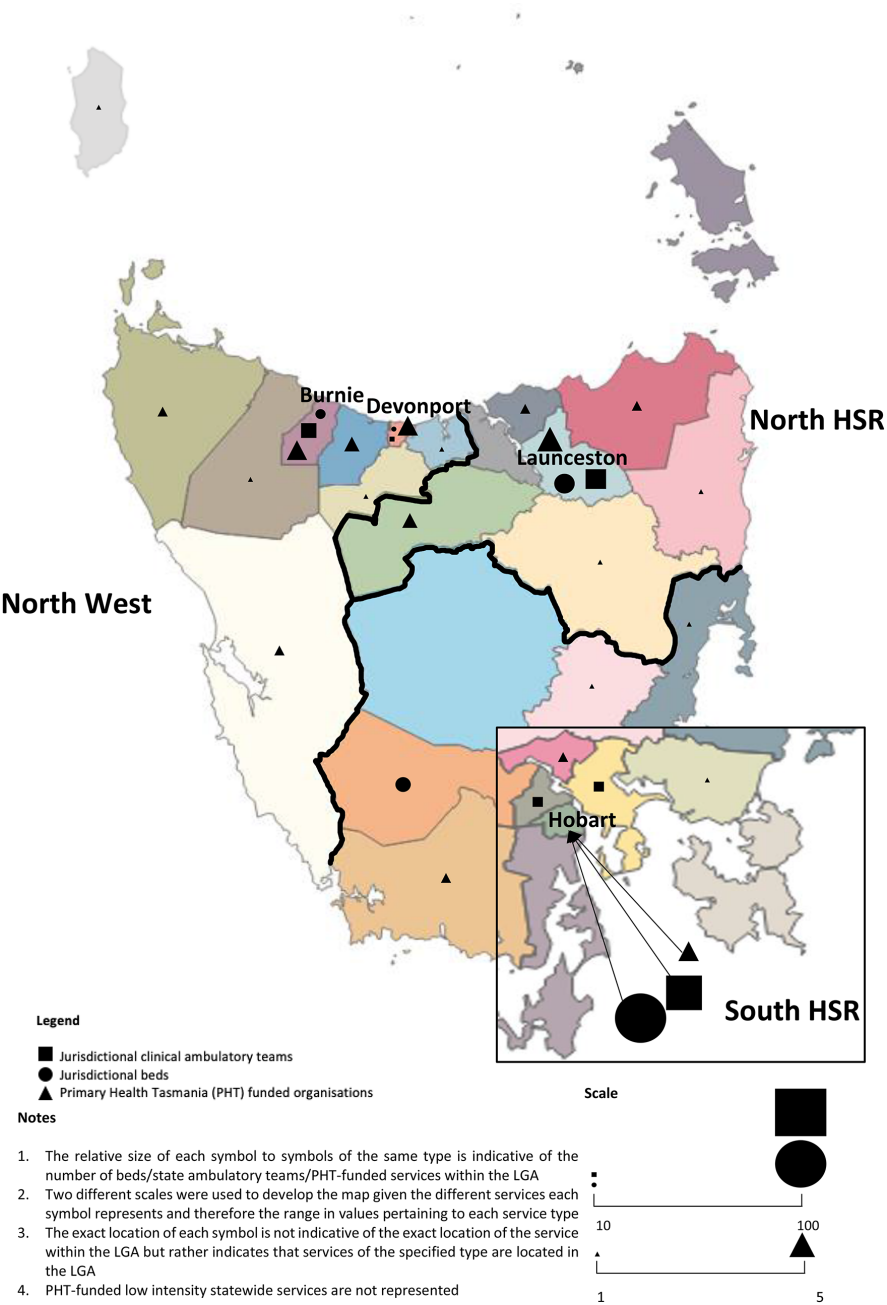
Location of mental health services across Tasmania, by Local Government Area (LGA)

Fig. 1 provides an overview of the location of mental health services across Tasmania in 2018-19. Findings indicated that the DoH was providing a range of acute, sub-acute and non-acute bed-based services. Acute bed-based services were located in all three HSRs while almost all (75%) of the sub-acute and non-acute bed-based services were located in the South HSR; however these were designated as state-wide services (i.e., they were available to all of Tasmania’s residents regardless of the area in which they resided). There were four private hospitals providing acute bed-based services in Tasmania; two in the South HSR and one in each of the North and North-West HSRs. There were a range of jurisdictional clinical ambulatory teams in Tasmania providing specialist care (i.e., child and adolescent, adult, and older adult clinical mental health teams, and crisis and assessment and consultation liaison teams). These clinical teams typically operated from a base in the major cities of each HSR.

Primary care services commissioned through PHT, excluding headspace centres, were mostly located in the North and North-West HSRs in 2018-19. headspace services for young people (12–25 years) were located across all three HSRs in the state’s most populous cities (i.e., Hobart, Launceston, Devonport).

In 2018-19, just over half (56%) of CMMHS provided by non-government organisations operated on a state-wide basis. Of the remaining services, 19% provided services in the North-West HSR, 16% in the South and 9% in the North.

### Access to services

**Fig. 2:**
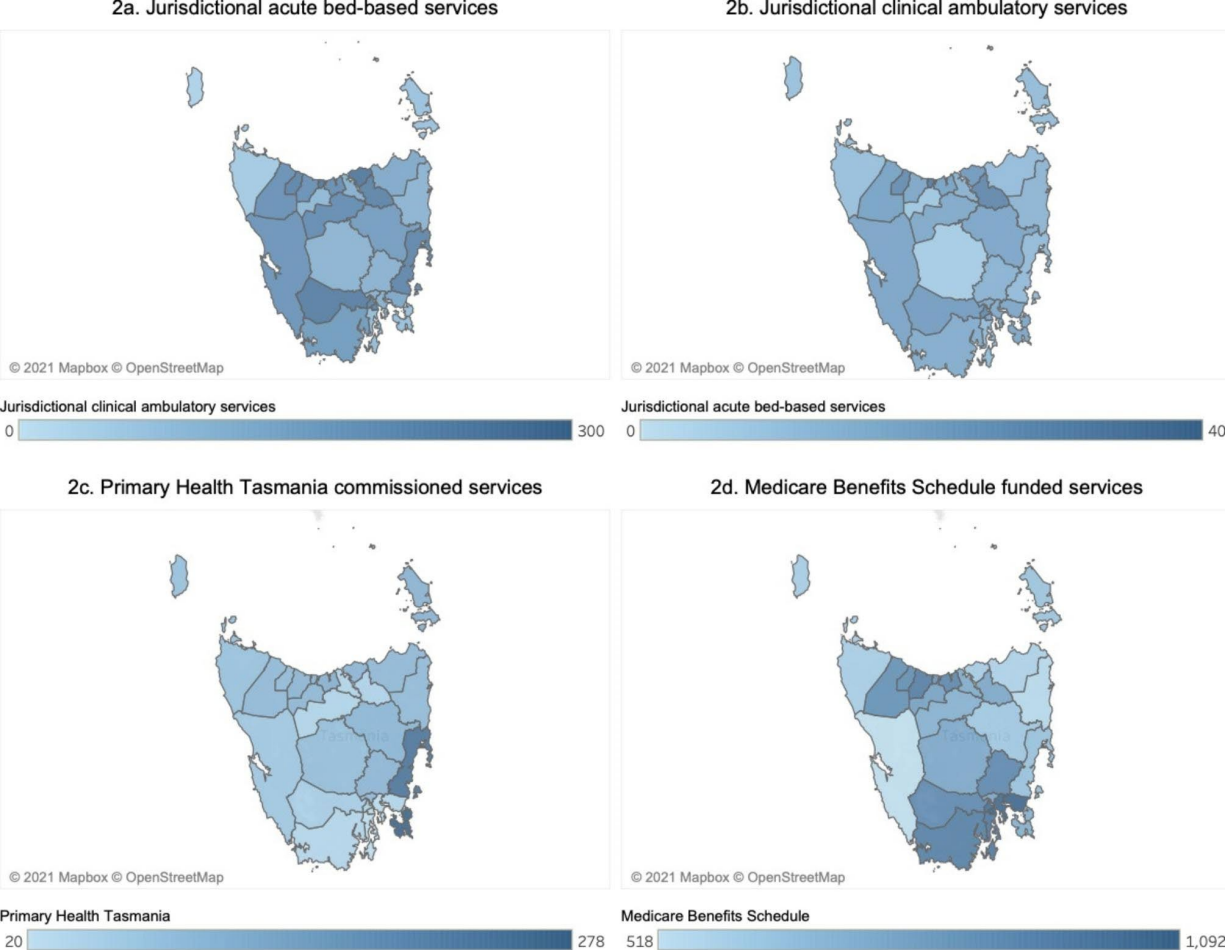
Access to mental health services across Local Government Areas, per 10,000 population

Rates of service access are shown in Fig. 2. Access to jurisdictional acute bed-based services was similar across HSRs (29, 28 and 24 per 10,000 population in the North-West, North and South respectively). However, when looking at the LGA level, two LGAs in the North-West HSR (Circular Head and King Island) had some of the lowest rates of access in the state (2–5 times lower than the HSR rate). Typically, in all HSRs access was higher in populous, urban LGAs and lower in more remote LGAs. An exception to this was Kingborough, an LGA close to Hobart LGA, which had one of the lowest rates of access in that HSR (2 times lower than the HSR rate for the South).

For jurisdictional clinical ambulatory services, rates of access were similar between the North-West and North HSRs (185 and 183 per 10,000 respectively) however access in the South was lower (155 per 10,000 population).

Rates of access to primary care and private clinical ambulatory services were examined separately for PHT and MBS services. For MBS services, access rates were highest in the South (1,013 per 10,000 population) followed by the North-West and North HSRs (857 and 725 per 10,000 population respectively). For PHT commissioned services, the opposite was true, with access rates higher in the North-West (100 per 10,000 population), compared to the North and South HSRs (58 and 45 per 10,000 population). There was no data pertaining to access to CMMHS as a whole; however, data from the NDIS showed that in 2019 around 60% of participants resided in the South [36].

### Activity and capacity

#### Bed based services

The average annual separation rate for the adult acute bed-based services was on par with the NMHSPF benchmark. The capacity of jurisdictional bed-based services (measured by number of available acute, sub- and non-acute beds) at the state-level reached 85% of the NMHSPF benchmark. Acute bed-based services were available for adult populations only (18–64 years) and capacity reached 89% of the NMHSPF benchmark. Sub-acute and non-acute bed-based services were available for adults and older adults (65 + years) and reached 76% and 85% of the NMHSPF estimates respectively. When looking at the capacity of bed-based services across HSRs, shortages in bed numbers were most prominent in the North and North-West HSRs (reaching 54% and 65% of the NMHSPF benchmark) and were due to the comparatively low number of sub-acute and non-acute beds in these regions. There were no bed-based mental health services of any type for children and young people in Tasmania at the time of this study.

#### Jurisdictional clinical ambulatory services

At the state-level, the capacity of jurisdictional clinical ambulatory services (measured as FTE staff counts) reached 58% of the NMHSPF benchmark. Regionally, the South had the greatest capacity (65%), followed by the North-West (62%) and North (46%). When considering jurisdictional clinical ambulatory teams across the state, the child and adolescent mental health teams, adult mental health teams and older adult mental health teams were reaching 61%, 62% and 53% of the NMHSPF benchmark for Tasmania. The capacity of the older adult mental health teams was consistently lower than the other age specific mental health teams across all three HSRs.

#### Primary care and private clinical ambulatory services

The collective activity (measured by occasions of service) occurring in state-level MBS, PHT, and headspace services reached 43% of the NMHSPF benchmark and capacity of these services (measured using total funding/expenditure) reached 57% of the NMHSPF benchmark.

#### Community managed mental health services

In terms of service capacity, funding provided to CMMHS approached the indicative costs estimated by the NMHSPF (90%).

## Discussion

This study provides an example of applying a needs-based planning model to inform mental health service development. It involved an assessment of existing resources and their utilisation to determine how future service design could ensure equitable service provision at the regional level. This was in alignment with the aims of Tasmania’s 2020 Rethink Strategy. Key findings related to each sector, and available indicators of service access, activity, and capacity, are discussed.

First, service access varied across and within HSRs. The North-West HSR had the highest rate of access to bed-based services. However, two LGAs in this region (Circular Head and King Island) had access rates among the lowest in the state, which may be explained by their remoteness [35]. Across all HSRs, rates of access to bed-based services were highest in urban regions, where most services were located, with some anomalies. For example, Kingborough LGA had one of the lowest rates of access to bed-based services in the state, despite its proximity to a major service area (Hobart LGA). Kingborough is a relatively advantaged area and its residents may be accessing private hospitals; Hobart Clinic, a private service, is located just outside the Kingborough LGA. Patterns in rates of access to jurisdictional clinical ambulatory services were similar. In the primary care and private clinical ambulatory service sector, rates of access to PHT commissioned services were highest in the Northern HSRs as compared to the South; the opposite was true for MBS services. This likely represents the strategic location of PHT commissioned services to account for the fewer private MBS providers in the Northern HSRs as compared to the South. Indeed, PHT’s health service directory suggests that a majority of these providers are located in the South HSR [37].

Second, available beds were used as an indicator of the capacity of bed-based services. The number of available beds was largely comparable to the NMHSPF estimate. The number of acute adult beds, and the average associated annual separation rate, were also on par with NMHSPF estimates. At the time of analysis, stakeholders revealed that Tasmania’s Hospital in the Home program was scheduled to commence in 2020, further increasing adult acute bed capacity across the state. Whilst there were fewer sub- and non-acute beds in the Northern HSRs as compared to the South HSR, the beds in the South HSR were state-wide services. These beds were, however, largely used by South HSR residents. Further investigation is needed to understand why residents in the Northern HSRs are not accessing these services (e.g., are they less inclined to use services away from their support networks? Are there other service types in the Northern HSRs catering to populations that require this type of care?). At the time of this study there were no dedicated child and adolescent mental health beds in Tasmania. Without access to age-specialist beds, young people in need are likely to either receive no inpatient treatment, or be admitted to adult units which increases risk of iatrogenic harm [38]. Notably, however, stakeholders mentioned the planned establishment of child and youth specific bed-based services that aim to fill this gap.

Third, the number of FTE staff employed in jurisdictional clinical ambulatory services was lower than the NMHSPF estimates, with this finding more pronounced in services for older persons. When interpreting these findings it should be acknowledged that the NMHSPF model carries assumptions about service operation (e.g., mix of professionals and hours worked in team-based services) and efficiency (e.g., the ratio of consumer-related time to other time such as travel, meetings etc.) that influence how estimates are produced. We ensured that on-the-ground FTE staff were defined and counted in a similar way to the NMHSPF to limit misinterpretation of the comparative analyses. The identified shortfalls in older persons FTE staff is not a new finding, nor is it unique to Tasmania. A recent inquiry noted that expansion in clinical ambulatory services across Australia is required to meet the needs of older persons [24]. Tasmania currently has the oldest population in Australia which, due to internal migration, is ageing faster than any other jurisdiction [29]. It is therefore expected that there will be continued increasing need for specialist services for older persons in Tasmania. Tasmania’s ‘Rethink 2020’ plan for mental health recognises that the recruitment of suitably qualified staff is one of the greatest challenges and is a key priority to meeting service demand [32].

Fourth, whilst funding/expenditure in the primary care and private clinical ambulatory services sector reached over half of the NMHSPF estimated indicative costs, activity (i.e. occasions of service) reached less than half of the NMHSPF estimate; there are several potential reasons for this finding. First, not all services in this sector were included in the comparative analysis (e.g. private health insurance, Department of Veterans’ Affairs and Worker’s Compensation funded ambulatory services). Additionally, even in included data collections there is the potential for missing data. For example, some mental health care provided by general practitioners is often not captured under the MBS mental health items included in the MBS data collection. Alternatively, lower than expected service activity may be representative of underservicing due to barriers to service access. If the latter is true, future service design should account for this potential barrier. For example, headspace outreach services have recently been established to increase access and uptake of primary mental health care services among disadvantaged young people in Tasmania [39].

Fifth, funding for CMMHS was largely comparable to the NMHSPF estimated indicative costs. Due to lack of data, it was not feasible to investigate the level of activity occurring within these services. Future work should examine the level and scope of activity occurring within these services to ensure that funding is used to deliver the full range of psychosocial support service types projected by the NMHSPF. Developing a better understanding of activity within this sector would also be helpful to further explore potential system imbalances. For example, it might be that areas with good provision of psychosocial support services see a reduction in the utilisation of bed-based services or the average length of treatment within state clinical ambulatory services.

### Limitations

This study had several limitations. First, data from some in-scope services were: (1) not routinely collected; or (2) not available for study purposes. Select indicators of service activity and capacity were therefore chosen for analysis based on the availability of on-the-ground services data. For example, for bed-based services, separations were used an as indicator of activity and available beds were used as an indicator of capacity. However, data on occupancy and staffing, which both have impacts on how services operate, were not available. Thus, a true like-for-like comparison for the bed-based services sector was not possible. Further, there were no routinely collected data for the CMMHS sector which meant that only a high-level comparative analysis of funding could be undertaken. Additionally, there were services data from ‘other’ primary care and private clinical ambulatory services that were not available for the purposes of this study. The inclusion of these data may have reduced the apparent activity and capacity gaps in this sector. However, these ‘other’ services are much smaller component of the overarching system compared to jurisdictionally funded specialist clinical services and MBS and PHN funded primary care services, as evidenced by the relatively smaller financial investment they receive [40]. Similarly, data pertaining to access, activity and capacity of privately funded bed-based services were also not available. This data may have provided contextual information to further inform the interpretation of findings regarding bed-based services.

Second, even when data existed, not all data of interest could be provided. For example, often service capacity data in the form of FTE staff counts was not routinely collected. For some data collections, we used funding/expenditure data instead. The ability to use a range of different types of activity and capacity data for comparison with the NMHSPF highlights the flexibility of the requirements of the model application. However, funding data included in the comparative analysis, specifically in the CMHHS sector, may have included costs that are out-of-scope for the NMHSPF model (e.g., capital costs). Furthermore, the quality of available data was variable across and within data collections and services. Outliers in the data often prompted discussions with custodians as to whether there were issues with the reliability of available data or the services themselves (e.g., were occasions of service low because of poor reporting, or was there genuine low activity within a service/across a collection of services), but it was not possible to quantify to what extent the data was reliable or otherwise within this study. Investigation is, however, warranted to determine whether there are issues with the data collections. If this is the case, concerted effort should be directed at improving reporting. It is suggested that ensuring professionals are aware of the purpose of data collection, and receive feedback based on an analysis of the collected data, may improve adherence to data collection protocols [24].

Third, V2.2 of the NMHSPF uses national average age-specific estimates of the prevalence of mental health problems and applies these to the population size and age distribution of different area levels to determine the level of need and associated resourcing requirements. However, socioeconomic features of areas and degrees of remoteness also contribute to variation in mental health service needs across regions [41]. According to the 2016 Census, Tasmania has the lowest proportion of people living in the most advantaged areas of all Australian states and territories and the highest proportion living in the most disadvantaged areas [42]. Additionally, within Tasmania, the South HSR comprises relatively fewer areas of disadvantage than the North and North West HSRs [43]. Much of the state is also considered outer regional or remote, while its two most populated cities, Hobart and Launceston, are classified as inner regional [35]. Thus, by applying national average estimates of service need to Tasmania, and the smaller regions that comprise it, this study is likely to underestimate levels of required resourcing and potential service gaps, particularly in the North and North West HSRs. Whilst estimates from the NMHSPF may be adjusted to better account for regional variation, this work was not part of the original project scope as it was not feasible within the project timelines.

Fourth, this study focused on understanding levels of mental health service capacity and activity from a population planning perspective. Health service utilisation is influenced by additional factors such as individual attitudes, stigma, and resource barriers including distance to and cost of services [44]. These are important further considerations in ensuring that people with mental health needs have equitable access to mental health services, however were beyond the scope of this analysis.

### Broader implications related to the application of needs-based planning models

This study has identified key considerations when mapping mental health services data and comparing these to the NMHSPF outputs. First, it has shown the importance of analysing services data at small area levels that are important for planning. Examining services data at larger area-levels (e.g., PHNs, LHNs or HSRs) may mask issues of service access, activity, and capacity at the local level at which planning decisions are made.

Second, our study highlights the importance of using contextual information to interpret comparative analyses with the NMHSPF. The NMHSPF is an important starting point for understanding resourcing requirements but should be supplemented with information on unique sociodemographic and contextual features that may influence local service access and need. Whilst local calibration of the NMHSPF model was considered from the outset of model development, a national average model was developed as an initial proof of concept with recognition of trade-offs between developing a locally calibrated model and further complicating the model. However, it should be acknowledged that development of the NMHSPF has since been completed to adapt its estimates for the specific mental health needs of Aboriginal and Torres Strait Islander and rural populations [45]. Additionally, NMHSPF licensed users and trainees have been surveyed approximately yearly since January 2022 with the intent of understanding user experience, including the facilitators and barriers to using the NMHSPF, how the NMHSPF is being used, and what improvements NMHSPF users and trainees want to see included in the model going forward. Findings from the current comparative analysis were contextualised by gathering information about existing services, services under development, and sociodemographic information for different geographical regions.

Third, this study has identified the importance of understanding the assumptions that underlie NMHSPF outputs. Specifically, a sound comparative analysis with the NMHSPF relies on the comparison of like-for-like services data. In this study we worked closely with the data custodians and service funders to ensure that on-the-ground services data was comparable to the NMHSPF estimates based on data definitions and the NMHSPF taxonomy.

Fourth, this work highlights the importance of developing a comprehensive map of all mental health services and collectively reviewing service location, access, activity, and capacity across service sectors. If services or sectors are examined in isolation, important information regarding system imbalances, or where one service type appears to fill the gap of another, may be missed.

Fifth, this study highlighted the value of buy-in from local stakeholders regarding application of the NMHSPF. Specifically, the inclusion of representatives from the PHN, State Department of Health, and community managed mental health support services ensured the following: (1) that data from these services were interpreted and contextualised correctly; (2) that existing services were accurately aligned with those modelled in the NMHSPF; (3) that recommendations for investment priorities based on the findings of the comparative analysis were feasible and considered all potential implementation issues; and (4) that a coordinated approach to implementing recommendations was understood by all service sector representatives. This study provided important information on services to help inform the State’s Rethink 2020 implementation plan [46].

## Conclusion

This study has shown that it is possible to use needs-based planning models like the NMHSPF to identify priority areas for service development. Whilst some study findings were limited by gaps in available data, this study provided a nuanced analysis and interpretation of how mental health service provision in Tasmania was tracking against the NMHSPF estimates of required care. It provides support for using needs-based planning models in this way and highlights issues for consideration when undertaking this type of work.

## Electronic supplementary material

Below is the link to the electronic supplementary material.

Supplementary Material 1: Mental health services data and associated analyses

## Data Availability

Services data analysed as part of this study are available from the Tasmania Department of Health (i.e., State-funded mental health services data), Primary Health Tasmania (i.e., PHN-commissioned services) and the Australian Institute of Health and Welfare (AIHW) (i.e., NMHSPF planning tool estimates and MBS summary data). Restrictions apply to the availability of these data, which were used under license for the current study and are therefore not publicly available. Data related to the NDIS was publicly available at the following link: https://data.ndis.gov.au/data-downloads.

## Abbreviations

DESDE-LTC: Description and Evaluation of Services and Directories in Europe for Long-Term Care
DoH: Tasmanian State government Department of Health
Fifth plan: Australia’s Fifth National Mental Health and Suicide Prevention Plan
HSR: Health Service Region
LGA: Local Government Area
LHN: Local Hospital Network
MBS: Medicare Benefits Schedule
NDIS: National Disability Insurance Scheme
NMHSPF: National Mental Health Service Planning Framework
PHN: Primary Health Network
PHT: Primary Health Tasmania
SA3: Statistical Area Level 3

## References

[1] Whiteford H, McKeon G, Harris M, Diminic S, Siskind D, Scheurer R (2014). System-level intersectoral linkages between the mental health and non-clinical support sectors: a qualitative systematic review. Australian & New Zealand Journal of Psychiatry.

[2] Wiktorowicz ME, Di Pierdomenico K, Buckley NJ, Lurie S, Czukar G (2020). Governance of mental healthcare: fragmented accountability. Soc Sci Med.

[3] Shen GC, Snowden LR (2014). Institutionalization of deinstitutionalization: a cross-national analysis of mental health system reform. Int J Mental Health Syst.

[4] Council of Australian Governments. The Fifth National Mental Health and Suicide Prevention Plan. Canberra; 2017.

[5] Australian Government Department of Health. Annexure A1 – Primary Mental Health Care 2016 [Available from: https://www1.health.gov.au/internet/main/publishing.nsf/Content/F4F85B97E22A94CACA257F86007C7D1F/$File/Annexure%20A1%20-%20Primary%20Mental%20Health%20Care.pdf.

[6] Australian Government Department of Health. Psychosocial support for people with severe mental illness 2020 [Available from: https://www1.health.gov.au/internet/main/publishing.nsf/Content/psychosocial-support-mental-illness.

[7] World Health Organization. Planning and budgeting to deliver services for mental health. Geneva 2003.

[8] Australian Institute of Health and Welfare. Health sector national minimum data sets 2020 [Available from: https://meteor.aihw.gov.au/content/index.phtml/itemId/344850.

[9] World Health Organization. Project Atlas 2020 [Available from: https://www.who.int/mental_health/evidence/atlasmnh/en/.

[10] Van Spijker BA, Salinas-Perez JA, Mendoza J, Bell T, Bagheri N, Furst MA (2019). Service availability and capacity in rural mental health in Australia: Analysing gaps using an Integrated Mental Health Atlas. Australian & New Zealand Journal of Psychiatry.

[11] Whiteford H, Diminic S, The National Mental Health Service Planning Framework (2020). Where has it come from and what is its future?. Australian & New Zealand Journal of Psychiatry.

[12] Woody C, Page I, Gossip K, John J, Wright E, Diminic S (2021). The National Mental Health Service Planning Framework – Service element and activity descriptions – commissioned by the australian Government Department of Health.

[13] Ritter A, Chalmers J, Gomez M. Measuring unmet deamnd for alcohol and other drug treatment: the application of an australian population-based planning model. J Stud Alcohol Drug. 2019:42–50. 10.15288/jsads.2019.s18.42 PMC6377016 30681948

[14] Australian Institute of Health and Welfare. National Mental Health Service Planning Framework, 2022 [Available from: https://www.aihw.gov.au/nmhspf.

[15] Rosen A, Rock D, Salvador-Carulla L (2020). The interpretation of beds: more bedtime stories, or maybe they’re dreaming?. Australian & New Zealand Journal of Psychiatry.

[16] Wright E, Leitch E, Fjeldsoe K, Diminic S, Gossip K, Hudson P et al. Using the National Mental Health Service Planning Framework to support an integrated approach to regional mental health planning in Queensland, Australia. Aust J Prim Health. 2021. 10.1071/PY20150 33653507

[17] Adelaide Primary Health Network. *Towards wellness: Adelaide metropolitan integrated mental health and suicide prevention plan*. 2020.

[18] Brisbane North Primary Health Network and Metro North Hospital and Health Service. *Planning for wellbeing: a regional plan for North Brisbane and Moreton Bay focusing on mental health, suicide prevention and alcohol and other drug treatment services 2018–2023*. 2018.

[19] Central and Eastern Sydney Primary Health Network. *Mental health and suicide prevention regional plan: Central and Eastern Sydney:2019–2022*. 2019.

[20] Western New South Wales Primary Health Network. Western NSW regional mental health and suicide prevention plan. 2020.

[21] Government of South Australia. Mental Health Services Plan 2020–2025. 2019.

[22] Mental Health Commission. Western australian Mental Health, Alcohol and other Drug Services Plan 2015–2025 update 2018. Government of Western Australia; 2019.

[23] Queensland Health. Connecting care to recovery 2016–2021: A plan for Queensland’s State-funded mental health, alcohol and other drug services. 2016.

[24] Australian Government Productivity Commission. Mental Health, Report no. 95. Canberra; 2020.

[25] State of Victoria. Royal commission into Victoria’s mental health system, final report (Parlimentary Paper No. 202, Session 2018–21). 2021.

[26] Whiteford H, Bagheri N, Diminic S, Enticott J, Gao CX, Hamilton M, et al. Mental health systems modelling for evidence-informed service reform in Australia. Australian & New Zealand Journal of Psychiatry; 2023. 10.1177/00048674231172113 37183347

[27] Rush B, Tremblay J, Brown D. Development of a needs-based planning model to estimate required capacity of a substance use treatment system. J Stud Alcohol Drug. 2019:51–63. 10.15288/jsads.2019.s18.51 PMC6377026 30681949

[28] Bruckner TA, Scheffler RM, Shen G, Yoon J, Chisholm D, Morris J (2011). The mental health workforce gap in low- and middle-income countries: a needs-based approach. Bull World Health Organ.

[29] Tasmanian Government. Population by age and sex, regions of Australia (ABS cat no 3235.0). Department of Treasury and Finance; 2019.

[30] Australian Bureau of Statistics. Income and work: Census 2022 [Available from: https://www.abs.gov.au/statistics/labour/earnings-and-working-conditions/income-and-work-census/2021.

[31] Australian Bureau of Statistics. National, state and territory population 2023 [Available from: https://www.abs.gov.au/statistics/people/population/national-state-and-territory-population/latest-release.

[32] Department of Health and Human Services. Rethink Mental Health: a long-term plan for mental health in Tasmania 2015–2025. Tasmanian Government 2015.

[33] Australian Bureau of Statistics. Population Projections, Australia 2017 [Available from: https://www.abs.gov.au/statistics/people/population/population-projections-australia/latest-release#key-statistics.

[34] The University of Queensland. NMHSPF - Taxonomy AUS V2.1. 2016.

[35] Australian Bureau of Statistics. The Australian statistical geography standard (ASGS) remoteness structure 2018 [Available from: https://www.abs.gov.au/websitedbs/d3310114.nsf/home/remoteness+structure.

[36] National Disability Insurance Scheme. Participant data 2019 [Available from: https://www.ndis.gov.au/about-us/data-and-insights/data/participant-data.

[37] Primary Health Tasmania. Tasmanian Health Directory - a directory of health providers in Tasmania 2021 [Available from: https://www.tashealthdirectory.com.au/specialties/1/home.

[38] McGorry PD (2007). The specialist youth mental health model: strengthening the weakest link in the public mental health system. Med J Aust.

[39] Bridgman H, Ashby M, Sargent C, Marsh P, Barnett T (2019). Implementing an outreach headspace mental health service to increase access for disadvantaged and rural youth in Southern Tasmania. Aust J Rural Health.

[40] Nous Group., Medibank. The case for mental health reform in Australia: a review of expenditure and system design. 2013.

[41] Enticott JC, Meadows GN, Shawyer F, Inder B, Patten S (2016). Mental disorders and distress: Associations with demographics, remoteness and socioeconomic deprivation of area of residence across Australia. Australian & New Zealand Journal of Psychiatry.

[42] Commissioner for Children and Young People (Tasmania) (2018). The Health and Wellbeing of Tasmania’s children and Young People Report 2018.

[43] Australian Bureau of Statistics. Socio-Economic Indexes for Australia (SEIFA), 2016 ‘Local Government Area, Indexes, SEIFA 2016 - Table 2. Local Government Area (LGA) Index of Relative Socio-economic Disadvantage, 2016’ 2018 [Available from: https://www.abs.gov.au/AUSSTATS/abs@.nsf/DetailsPage/2033.0.55.0012016?OpenDocument.

[44] Anderson RM (1995). Revisiting the behavioural model and access to medical care: does it matter?. J Health Soc Behav.

[45] Diminic S, Gossip K, Page I, Woody C (2021). Introduction to the National Mental Health Service Planning Framework - commissioned by the australian government.

[46] Primary Health Tasmania and the Tasmania Department of Health. Rethink 2020: A state plan for mental health in Tasmania 2020–2025. 2020.

