# Supplementary material for: Using the National Mental Health Service Planning Framework to inform integrated regional planning: a case study in Tasmania, Australia
Source: Int J Ment Health Syst. 2023 Jul 22;17:23. doi: 10.1186/s13033-023-00591-w (PMC10362704; doi:10.1186/s13033-023-00591-w)
Supplement: Supplementary file 1 — Supplementary Material 1: Mental health services data and associated analyses [file 13033_2023_591_MOESM1_ESM.docx]

Supplementary material for Using the National Mental Health Service Planning Framework to inform integrated regional planning: a case study in Tasmania, Australia.

Supplementary Table 1. Mental health services data and associated analyses

| **Services data** | | | | | |  | **Data analysis** | | | |
| --- | --- | --- | --- | --- | --- | --- | --- | --- | --- | --- |
|  |  |  |  |  |  |  | **Transformation methods to align services data with NMHSPF outputs** | **Key analyses** | | |
| **Service sector** | **Data custodian** | **Data source** | **Service type** | **Data year^$^** | **Available data** |  |  | **Service access** | **Service activity** | **Service capacity** |
| Bed-based services | Tasmanian DoH | Internal Tasmanian DoH records | Tasmanian DoH funded bed-based mental health services (i.e., hospital admitted and residential services) | 2018 – 19 | - No. of consumers - Consumer usual area of residence - Available beds - Bed type |  | Bed types were aligned with those represented in the NMHSPF | No. of consumers by consumer usual area of residence (only available for consumers of acute beds-based services) | Separations (only available for consumers of acute bed-based services) | Available beds by bed type (acute, sub-, and non-acute) |
|  | Private hospitals | Internal private hospital records | Privately funded bed-based services | 2018 – 19 | Data unavailable for project |  | NA | NA | NA | NA |
| Jurisdictional clinical ambulatory services | Tasmanian DoH | Internal Tasmania DoH records | Tasmanian DoH funded clinical ambulatory mental health services | 2018 – 19 | - No. of consumers - Consumer usual area of residence - FTE staff counts - Treatment team type |  | Treatment team types were aligned with those represented in the NMHSPF | No. of consumers by consumer usual area of residence | NA | FTE staff counts by treatment team type |
| Primary care and private clinical ambulatory services | Australian Prudential Regulation Authority^%^ | Publicly reported private health insurance membership and benefits data | Private health insurance funded clinical ambulatory mental health services provided by a psychologist | 2018 – 19 | - Occasions of service - Fees paid |  | Nil | Data was only available at the state level and an analysis of service access could not be undertaken. | Occasions of service | Fees paid |
|  | Department of Veterans Affairs (DVA) | DVA centrally held person-level services data | Clinical ambulatory services funded by DVA | 2018 – 19 | Data unavailable for project |  | NA | NA | NA | NA |
|  | headspace^§^ | Annual headspace report for Primary Health Tasmania | Non-Medicare Benefits Schedule funded clinical ambulatory services‘ | 2018 - 19 | - No. of consumers - Service location - Occasions of service - Funder - Funding |  | The proportions of occasions of service that were not funded by the Medicare Benefits Schedule were estimated for each service location | No. of consumers by consumer usual area of residence | Occasions of service (non-MBS funded) | Funding |
|  | Primary Health Tasmania“ | Primary Mental Health Care Minimum Data Set | Clinical ambulatory services commissioned by Primary Health Tasmania | 2018 - 19 | - No. of consumers - Consumer usual area of residence - Occasions of service - Funding |  | Postcode data was aggregated to the LGA level using ABS concordance files and contact types were aligned with NMHSPF service elements | No. of consumers by consumer usual area of residence | Occasions of service | Funding |
|  | MindSpot Clinic^†^ | Centrally held services data | Clinician-led/moderated web-based services | 2018 - 19 | - No. of consumers - Consumer usual area of residence - National average estimate of occasions of service per consumer, per service contact type |  | National average estimates of occasions of service per consumer, per service contact type, were multiplied by the number of consumers in each LGA to estimate service activity | No. of consumers by consumer usual area of residence | Occasions of service by consumer usual area of residence | NA |
|  | The Australian Institute of Health and Welfare | MBS-NMHSPF mapped data^‡^ | Mental health specific services funded by the Medicare Benefits Schedule | 2015 - 16 | - No. of consumers - Consumer usual area of residence - Occasions of service - Expenditure |  | SA3 data was aggregated to the LGA-level using ABS concordance files | No. of consumers by consumer usual area of residence | Occasions of service by consumer usual area or residence | Expenditure |
|  | THIS WAY UP | Centrally held services data | Clinician-led/moderated web-based services | 2018 - 19 | - No. of consumers - Consumer usual areas of residence - Occasions of service per service contact type |  | Nil | No. of consumers by consumer usual area of residence | Occasions of service by consumer usual area of residence | NA |
|  | SafeWork Australia | National Dataset for Compensation Based Statistics | Workers Compensation funded mental health service data |  | Data unavailable for project |  | Nil | NA | NA | NA |
| Psychosocial support services | Primary Health Tasmania | Primary Mental Health Care Minimum Data Set | Psychosocial support services commissioned by Primary Health Tasmania^¶^ | 2019 - 20 | - Service description - Service location - Funding |  | Service descriptions/contact types were aligned with NMHSPF service elements | NA | NA | Funding |
|  | Tasmanian DOH | Internal service contracting records | Tasmanian DoH funded psychosocial support services^^^ | 2017 - 18 | - Service description - Service location - Funding |  | Service descriptions/contact types were aligned with NMHSPF service elements | NA | NA | Funding |
|  | Commonwealth Department of Social Services | Internal service contracting records | Commonwealth funded psychosocial support services^\|^ | 2016 - 20 | - Service description - Service location - Funding |  | Estimated average annual funding was calculated by dividing program funding amount by the duration of funding and service descriptions were aligned with NMHSPF service elements | NA | NA | Funding |
|  | National Disability Insurance Agency | National Disability Insurance Scheme publicly reported consumer level data | Services defined as ‘core’ or ‘capacity’ supports provided to consumers with a psychosocial disability^\|\|^ | June 2019 quarter | - No. of consumers - Consumer usual area of residence - Support class description - Funding (i.e. average annual committed budget plan) - National average plan utilisation estimate for consumers with psychosocial disability |  | No. of consumers was multiplied by average committed plan budgets to determine total average committed expenditure and a national average utilisation estimate was applied to the resulting figures to estimate the total average utilised expenditure | No. of consumers by consumer usual area of residence | NA | Expenditure |

*NMHSPF: National Mental Health Service Planning Framework; DoH: Department of Health; FTE: Full-time equivalent; FY: financial year; NA: Not available; SA3: Statistical Area Level 3; LGA: Local Government Area; ABS: Australian Bureau of Statistics*

**^$^** *Refers to financial year*

^%^ *Data not included in analysis*

^§^ *More detailed headspace services data is available via headspace’s national office. However, these data were unavailable for the project.*

‘ *headspace service activity that is funded by the Medicare Benefits Schedule (MBS) is already captured in the Australian Institute of Health and Welfare’s NMSHPF-MBS mapped dataset. For this reason, we were only interested in analysing non-MBS funded service activity and capacity data from this data source.*

“ *Refers to the following service contact types: psychological assessment, structured psychological interventions, other psychological interventions, clinical care coordination and liaison, clinical nursing services, child and youth assistance not otherwise classified (NEC), suicide prevention specific assistance NEC.*

^†^ *Due to lack of expenditure data, these data were not included in the analysis of activity and capacity for the primary care and private clinical ambulatory services sector*

^‡^ *The MBS-NMHSPF mapped dataset comprises Statistical Area Level 3 (SA3) activity and capacity data pertaining to in-scope MBS items. In-scope items are those that align with the outputs of the NMHSPF. This data source excludes activity and capacity data pertaining to care provided by a general practitioner not classified under MBS mental health items*

^¶^ *Refers to any services commissioned by Primary Health Tasmania that collect activity data defined by the service contact type ‘psychosocial support’*

^^^ *Refers to community-based recovery and rehabilitation programs, home-based support services, vocational rehabilitation services, and community-based social activities and groups*

^|^ *Respite care for carers of people with mental illness, Individual Placement Support for young people (up to 25 years of age), support to vulnerable families with children and young people (0 – 18 years of age) who are showing early signs or are at risk of developing mental illness*

^||^ *NDIS ‘core’ supports are defined as ‘assistance with activities of daily living‘ and ‘capacity’ supports are defined as assistance with, for example, increasing social and community participation, finding and keeping a job, and improving living arrangements. Core and capacity supports are the only supports under the NDIS that align with those represented in the NMHSPF*
